# Gamma-hexalactone flavoring causes DNA lesion and modulates cytokines secretion at non-cytotoxic concentrations

**DOI:** 10.1186/s40360-019-0359-x

**Published:** 2019-12-19

**Authors:** Luísa Zuravski, Taiane A. Escobar, Elizandra G. Schmitt, Queila D. F. Amaral, Fávero R. Paula, Thiago Duarte, Marta M. M. F. Duarte, Michel M. Machado, Luís F. S. Oliveira, Vanusa Manfredini

**Affiliations:** 1 Programa de Pós-Graduação em Bioquímica, Universidade Federal do Pampa, Uruguaiana, Brazil; 2 Curso de Farmácia, Universidade Federal do Pampa, Uruguaiana, Brazil; 3 Programa de Pós-Graduação em Ciências Farmacêuticas, Universidade Federal do Pampa, Uruguaiana, Brazil; 4 Programa de Pós-Graduação em Farmacologia, Centro de Ciências da Saúde, Universidade Federal de Santa Maria, Santa Maria, Brazil; 5 Universidade Luterana do Brasil, Santa Maria, Brazil

**Keywords:** Flavoring, γ-Hexalactone, Lymphocytes, In silico, In vitro

## Abstract

**Background:**

The γ-hexalactone is a flavoring agent for alcoholic beverages, teas, breads, dairy products, coffees, buttery products among others. It presents low molecular weight and exhibits sweet fruity aroma with nuances of nuts. As far as we know, both literature and government regulations have gaps regarding the safe use of the γ-hexalactone. In this context, the main objective of this work was to evaluate the effects of γ-hexalactone through in silico and in vitro approaches.

**Methods:**

The in silico analysis was performed through four free online platforms (admetSAR, Osiris Property Explorer^®^, pkCSM platform and PreADMET) and consisted of comparative structural analysis with substances present in databases. The computational prediction was performed in the sense of complement and guide the in vitro tests. Regarding in vitro investigations, screening of cytotoxicity (assessed by cell proliferation and viability parameters) in lymphocytes exposed to γ-hexalactone for 72 h were carried out previously to determine non-cytotoxic concentrations. Following this screening, concentrations of 5.15, 0.515, and 0.0515 μM were selected for the study of the respective potentials: genotoxic (assessed by DNA comet assay), chromosomal mutation (analysis of micronucleus frequency) and immunomodulatory (cytokine quantification using ELISA immunoassay). The results of in vitro assays were compared by one-way analysis of variance (ANOVA), followed by Bonferroni’s post hoc test, conducted by statistic software.

**Results:**

The platform PreADMET pointed out that γ-hexalactone is potentially mutagenic and carcinogenic. The comet assay data corroborate with these results demonstrating that γ-hexalactone at 5.15 μM caused lymphocytes DNA damage. In relation to cytokine secretion, the results indicate that lymphocytes were activated by γ-hexalactone at non-cytotoxic concentrations, involving an increase in the IL-1 levels in all tested concentrations, ranging from approximately 56 to 93%. The γ-hexalactone only at 5.15 μM induced increase in the levels of IL-6 (~ 60%), TNF-α (~ 68%) and IFN-γ (~ 29%), but decreased IL-10 (~ 46%) in comparison with the negative control (*p* < 0.05). No change was observed in total lymphocytes or in cell viability at the concentrations tested.

**Conclusions:**

In summary, the γ-hexalactone demonstrated immunomodulatory and genotoxic effects at non-cytotoxic concentrations in healthy lymphocytes.

## Background

The γ-hexalactone improves the aroma and/or flavor of various food preparations such as alcoholic beverages, teas, breads, dairy products, coffees, buttery products among others [1]. Moreover, it is suitable to be used as fragrances in personal care (perfumes, creams, etc.) and household products [2].

The Food and Drug Administration (FDA) approved the γ-hexalactone for food use and the Council of Europe included it at a level of 10 ppm (10 μg/mL) in the list of artificial flavoring substances that may be added temporarily to foodstuffs without hazard to public health [1]. Moreover, Joint Food and Agriculture Organization (FAO) of the United Nations/World Health Organization (WHO) Expert Committee on Food Additives (JECFA) recommends the human safe intake threshold is 1800 μg/person/day [3].

However, the true or real human exposure to γ-hexalactone is difficult to predict, because there is no complete information regarding the of its use and the differs way exposure. In addition, toxicological activities such as cytotoxicity and mutagenicity risk are not well known. In this context, this study aimed to evaluate the γ-hexalactone effects through computational and in vitro toxicological approaches.

## Materials and methods

### Chemicals

γ-Hexalactone (CAS number 695–06-7), histopaque-1077^®^ and reagents for cell culture, including RPMI 1640 medium, fetal bovine serum (FBS), penicillin/streptomycin, phytohemagglutinin-M (PHA-M) were purchased from Sigma-Aldrich (St. Louis, MO, USA). All other chemicals were of analytical grade and obtained from standard commercial suppliers.

### In silico toxic risk prediction

Four online computer programs were employed to estimate the possible toxicity risks of γ- hexalactone: admetSAR server [4], Osiris Property Explorer^®^ [5], pkCSM platform [6] and PreADMET [7]. The mutagenicity and carcinogenicity effects were interpreted and expressed as “yes” or “no” and “not-detected” risk.

### In vitro toxicological assays

#### Peripheral blood mononuclear cells (PBMC) purification

Whole fresh human blood (20 mL) was collected (ethics protocol in Universidade Federal do Pampa n° 27,045,614.0.0000.5323) into heparinized vacutainers by venipuncture from healthy adult volunteers (*n* = 3; 20–35-year-old, non-smokers, nonalcohol consuming and not undergoing any medication). Briefly, fresh blood was transferred to conical centrifuge vials containing Histopaque-1077^®^ density gradient centrifugation (1:1). The conical tube was centrifuged for 30 min at 400×g and PBMC were positioned in the interface. The opaque interface containing PBMC was carefully aspirated and then transferred into a clean conical centrifuge tube. The cells were washed by adding phosphate buffered saline (PBS) and centrifuged at 250×g for 10 min. The pellet was resuspended with RPMI 1640 medium. The cell viability using trypan blue dye exclusion method and cell count were assessed in a Neubauer’s hemocytometer under optical microscopy. The PBMC suspension presented viability of 98%. All the experiments were performed in triplicate.

#### Lymphocytes culture

A cell suspension comprising 10^6^ PBMC was cultivated in RPMI 1640 culture medium supplemented with 10% FBS, penicillin (100 U/mL), and streptomycin (100 mg/mL). PHA-M at 1 mg/mL was added to stimulate the human peripheral blood lymphocytes. Negative control in all the experiments consisting of lymphocytes suspended in RPMI-1640 medium and the positive control contained 2.12 μM bleomycin. Both were processed in the same way as the treatment cultures, but without γ-hexalactone. All assays were performed in triplicate from independent experiments.

#### Cytotoxicity screening

The concentrations selection for the present study was based on initial cytotoxicity screening. For this, human lymphocytes were exposed to γ-hexalactone at a range of concentrations (876.12 μM - 0.087612 μM) and incubated at 37 °C in 5% CO_2_ for 72 h, as described in a previous work by our group [8]. After this period, total lymphocytes were counted in a Neubauer’s hemocytometer and IC_50_ (50% cell-growth inhibition) values were calculated by a nonlinear regression method (*data not shown*). Following the initial screening, the concentrations 5.15, 0.515 and 0.0515 μM, which represent values of IC_50/10_, IC_50/100_, and IC_50/1000_ respectively, were used for studying the toxicological profile of γ-hexalactone in lymphocytes. According to FAO/WHO [9] 10 to 20% of the intake amount can reach the bloodstream, so the range of concentrations tested in our study may represent actual values of exposure to this flavoring.

#### Cellular proliferation assay

The cell culture was previously evaluated using a Neubauer’s hemocytometer [8]. The total cells count was performed, and the results were presented as total lymphocytes per culture flask.

#### Cellular viability assay

The cytotoxicity analysis was performed by trypan blue dye exclusion method [10] which is based on the loss of the integrity of the cell membrane. In this method, briefly, 25 μL lymphocyte’s cultures were exposed to trypan blue dye (0.4% w/v) and, after 3 min, an aliquot was placed in a Neubauer chamber under a microscope at a magnification of 400× for the differential analysis. The viable cells are impermeable to the dye whereas non-viable cells, due to the formation of pores in the membrane, are permeable to the dye and thus exhibit a blue color. The results are presented as the percentage of living cells (i.e., those not stained with trypan blue) [11–13].

#### Micronucleus test

The micronucleus test was performed according to the technique described by Schmid [14]. After the incubation period, the lymphocytes suspension was harvested for slide preparation. The slides were stained with rapid commercial hematologic staining (New Prove^®^, Brazil), analyzed by at least two different individuals who were blinded to the conditions and the mean of the two evaluators was used. For each slide, 200 lymphocytes were scored and classified according to absence or presence of the micronuclei.

#### Comet assay

The comet assay was performed according to Singh et al. [15]. After the incubation period, the lymphocytes were suspended in low-melting-point agarose and spread onto a glass microscope slide. Dry slides were incubated in ice-cold lysis solution (2.5 M NaCl, 100 mM EDTA, 10 mM Tris, pH 10.0 and 1% Triton X-100 with 10% DMSO). After lysis, slides were placed on a horizontal electrophoresis unit, covered with a fresh solution (300 mM NaOH and 1 mM EDTA, pH > 13). Electrophoresis was performed for 20 min (25 V; 300 mA). Slides were then neutralized, washed, and stained with 0.1% AgNO_3_. Slides were analyzed using an optical microscope. One hundred cells from each of the three replicate slides were analyzed by at least two different individuals who were blinded to the conditions. Cells were visually scored according to tail length and receive scores from 0 (no migration) to 4 (maximal migration) according to tail length. Therefore, the damage index for cells ranged from 0 (all cells with no migration) to 400 (all cells with maximal migration).

#### Cytokines quantifications

The levels of the cytokines IL-1, IL-6, TNF-α, IFN-γ and IL-10 were measured in cell-free supernatants using ELISA immunoassay kits according to the manufacturer’s instructions (Neogen do Brasil, Indaiatuba, SP, Brazil) [16]. The results were expressed as cytokine unit measures (pg/mL).

### Statistical analysis

IC_50_ was calculated employing nonlinear regression in GraphPad Prism 7 software [17]. For all other assays, the Gaussian distribution was verified by the Kolmogorov-Smirnov test. After that, significant differences were determined using one-way analysis of variance (ANOVA) followed by Bonferroni’s post hoc conducted by the same software. The results were expressed as mean ± SD and *p* < 0.05 was considered as statistically significant.

## Results

### In silico toxic risk prediction

According to PreADMET program (Table 1) the γ-hexalactone presents potential mutagenic and carcinogenic.

**Table 1:** Toxicity risk prediction for the γ-hexalactone from the computer simulation

ID substance	Toxic risk by admetSAR ^1^; Osiris Property Explorer ^2^, pkCSM ^3^; PreADMET ^4^;	
	Mutagenic	Carcinogenic*
γ-hexalactone	Not Detected ^1^ Not Detected ^2^ Not Detected ^3^ Yes ^4^	Not Detected ^1^ Not Detected ^2^ Yes ^4^

The toxic risks assessed, mutagenicity and carcinogenicity side effects were interpreted and expressed as “Yes” or “No” and “Not Detected” risk. *The pkCSM platform does not evaluate the carcinogenicity parameter. The superscripted numbers identify the platform used in the predictions

### In vitro toxicological analyses

#### Cellular proliferation assay

**Fig. 1:**
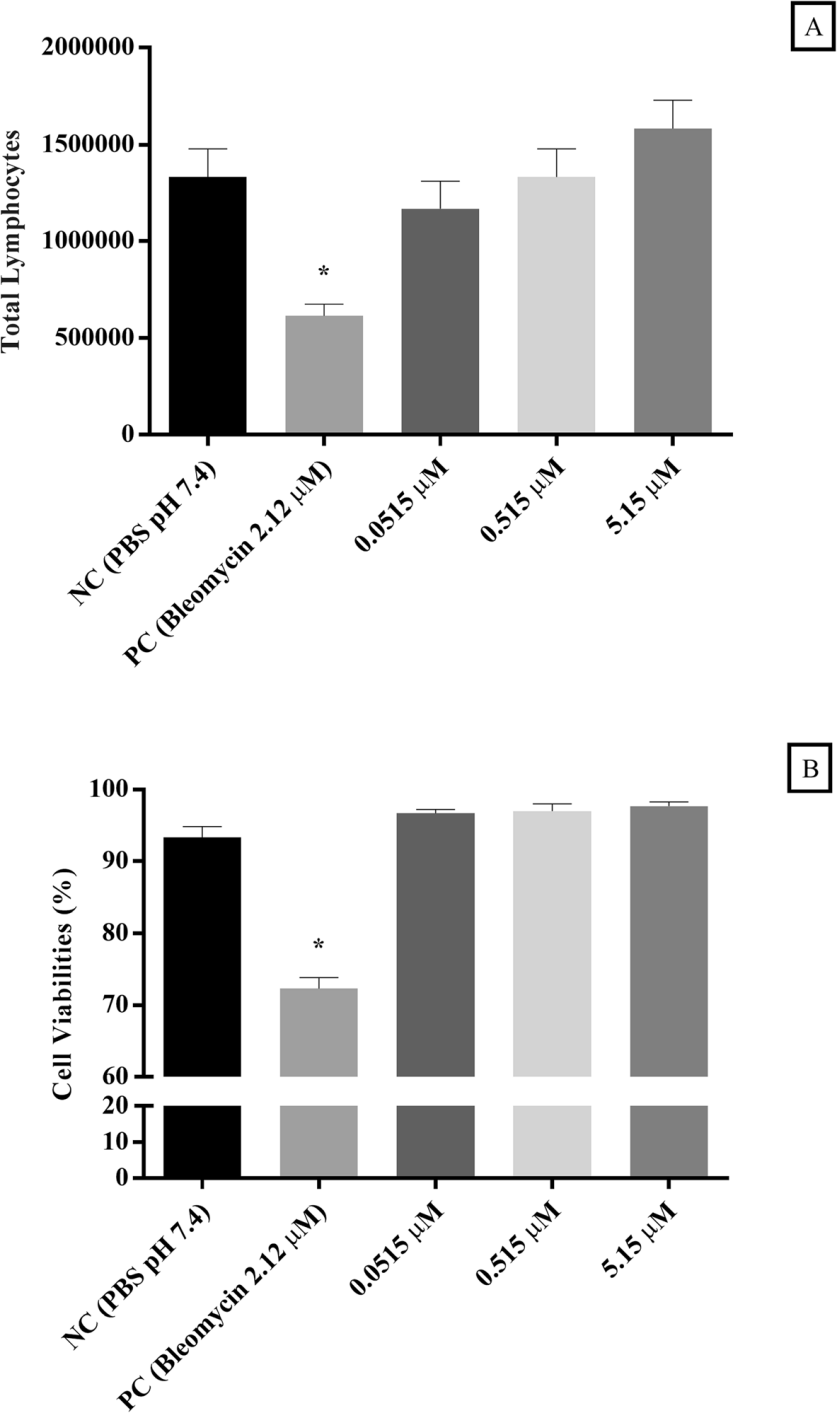
Evaluation of cell proliferation and viability in cultured lymphocytes (**a** and **b**) exposed to different concentrations of γ-hexalactone. Data were expressed as mean ± standard deviation, n = 3, and analyzed by one-way analysis of variance (ANOVA) followed by Bonferroni’s post hoc; (*) represent a statistically significant difference at *p* < 0.05 in relation to the negative control (NC)

The total number of cells after exposure to non-cytotoxic concentrations of γ-hexalactone remained like the negative control group (Fig. 1a).

#### Cellular viability assay

The lymphocytes showed viability greater than 96%, after exposure to γ-hexalactone at non-cytotoxic concentrations for 72 h (Fig. 1b).

#### Micronucleus test

**Fig. 2:**
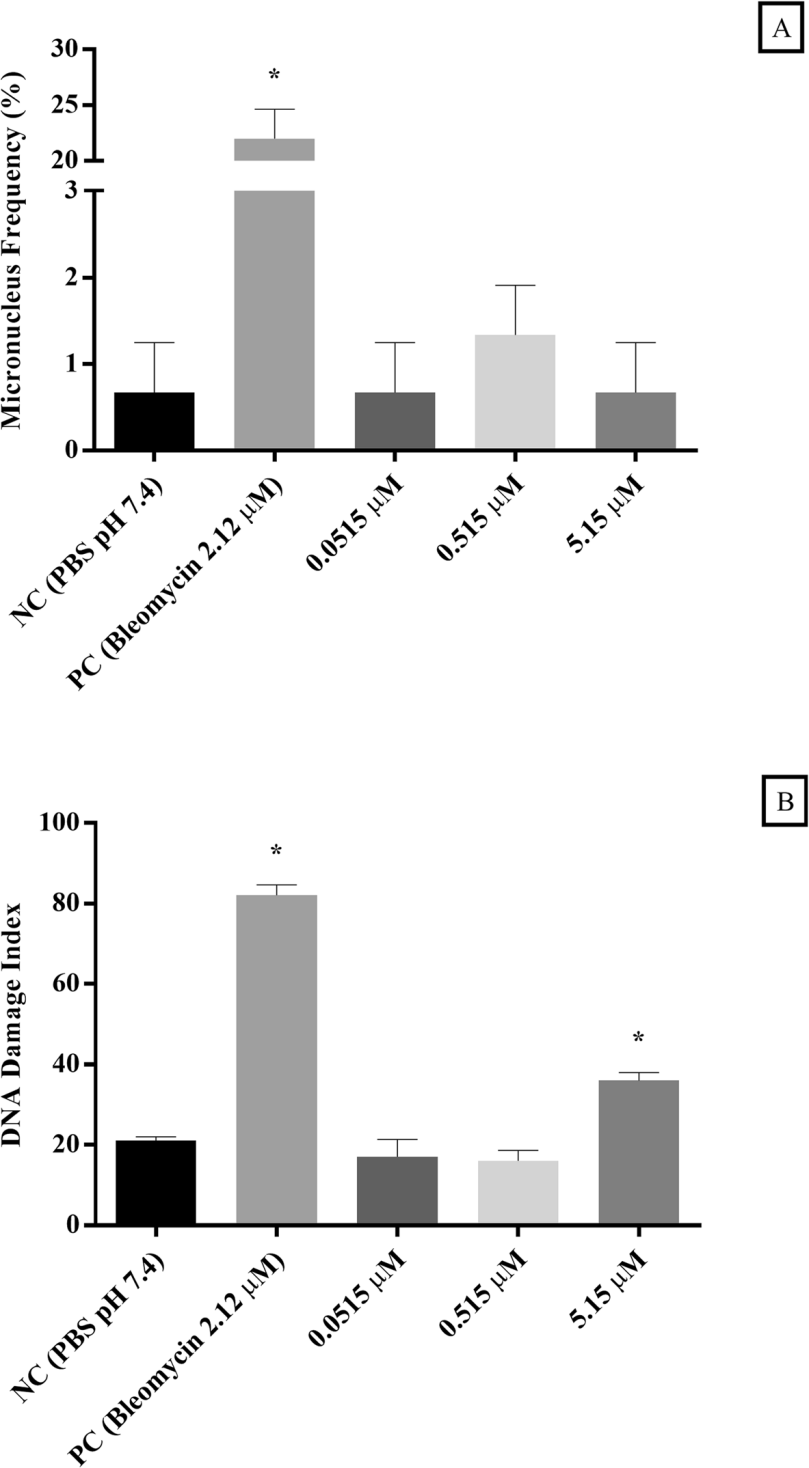
Micronucleus frequency and DNA damage index in cultured lymphocytes (**a** and **b**) exposed to different concentrations of γ-hexalactone. Data were expressed as mean ± standard deviation, n = 3, and analyzed by one-way analysis of variance (ANOVA) followed by Bonferroni’s post hoc; (*) represent a statistically significant difference at *p* < 0.05 in relation to the negative control (NC)

Figure 2a reports that γ-hexalactone did not induce micronucleus formation under the experimental conditions and concentrations assayed in human lymphocytes culture, when compared with the negative control (*p* < 0.05).

#### Comet assay

The Fig. 2b shows that γ-hexalactone at 5.15 μM, induced a DNA damage in the cultured lymphocytes reaching 71.43% higher than negative control (*p* < 0.05).

#### Cytokine quantification

**Fig. 3:**
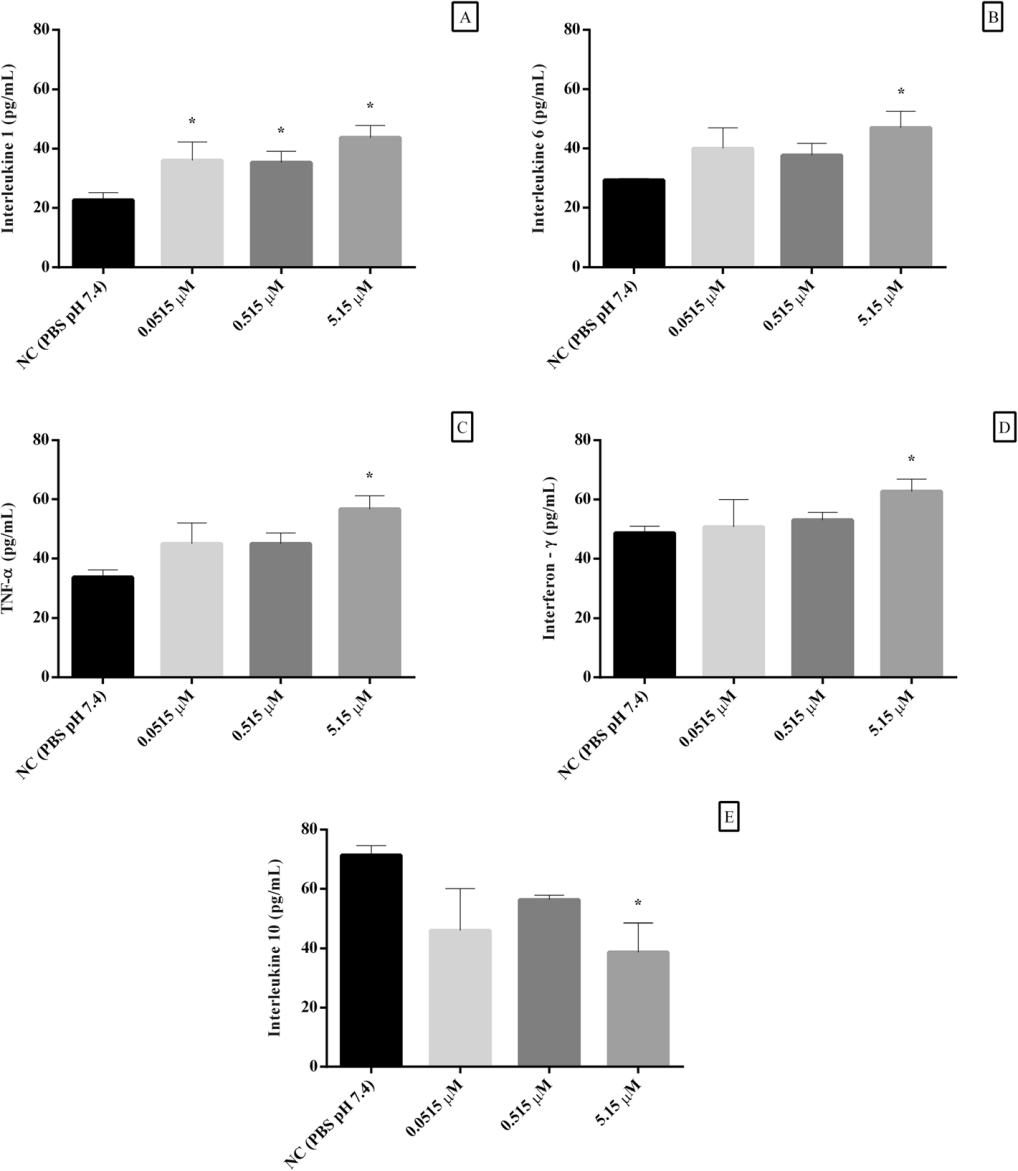
The cytokine levels (IL-1 (**a**), IL-6 (**b**), TNF-α (**c**), IFN-γ (**d**) and IL-10 (**e**)) at lymphocytes exposed to different concentrations of γ-hexalactone. Data were expressed as mean ± standard deviation, n = 3, and analyzed by one-way analysis of variance (ANOVA) followed by Bonferroni’s post hoc; (*) represent a statistically significant difference at *p* < 0.05 in relation to the negative control (NC)

Figure 3 shows the cytokine levels from the cultured lymphocytes exposure to non-cytotoxic concentrations of γ-hexalactone. This additive significantly increased the IL-1 levels in all tested concentrations, ranging from approximately 56 to 93% in comparison with the negative control (*p* < 0.05). The γ-hexalactone at 5.15 μM induced increase the levels of IL-6 (~ 60%), TNF-α (~ 68%), and IFN-γ (~ 29%), but decreased the IL-10 (~ 46%).

## Discussion

The present study investigated the effect of flavoring food γ-hexalactone through in silico and in vitro approaches. PreADMET platform pointed out that γ-hexalactone is potentially mutagenic and carcinogenic. On the other hand, γ-hexalactone was unable to induce chromosomal mutation under the experimental conditions assayed in human lymphocytes culture. The differences between in silico and in vitro results may be supported by the use of different methodologies, once the first one predicted mutagenic potential based in data banks of Ames test using *Salmonella typhimurium* strains [7]; and the second, by micronucleus test. In the in silico tests, the models try to assess the compounds effects or its toxicity using accurate molecular comparisons with different endogenous molecular targets. In vitro tests try to reproduce an environment to mimic a realistic cellular exposure to compounds of interest, including the parameters as concentration range and exposure time [18]. Although these limitations exist, the in silico methodology has shown to be relevant to delineate studies and to serve as a method to explain the phenomena of the mechanisms of action. Moreover, in silico evaluations are advantageous for the rapid execution, low cost, and the ability to reduce the animal use in toxicity testing [19]. We must consider that the predictions methods are based on comparative evaluation of test molecules with databases and therefore, they are not always full reliable evaluations, requiring more than one platform for each parameter assayed or experimental validations like those performed here. Thus, the in silico findings can be either a complement or a screening for subsequent tests, but not replace the in vitro or in vivo analysis.

Regarding the genotoxicity, the comet assay revealed significant DNA damage in the lymphocytes exposed to γ-hexalactone at 5.15 μM concentration in comparison with the negative control. Although we have presented in Table 1 only the mutagenicity and carcinogenicity data, the platforms provide additional information, which may be useful in the exploration of mechanisms of action. In our study, DNA damage may be related to the prooxidant status of γ-hexalactone defined by the presence of two electron accepting sites in its structure according to the pKCSM platform. The ability to accept electrons generates reactive species that may be involved in the initiation and propagation of chain reactions with macromolecules and consequent cell damage [11, 20]. Sinha and co-workers demonstrated that higher than 25 μg/mL concentration of citral, a flavoring widely used in food, also induced genotoxicity in human lymphocytes [11]. Further, the DNA damage may be connected to the carcinogenic potential indicated by computational analysis (PreADMET).

Cytokines such as IL-1, IL-6, TNF-α, IFN-γ, and IL-10 are biomarkers to assess the immune cell function because their production or secretion are linked to activation, differentiation, inflammatory, and apoptosis in immune system responses [21]. In our experiments, we observed that γ-hexalactone modulates the lymphocytes cytokines, increasing the IL-1, IL-6, TNFα, and IFN-γ levels, while decreasing the IL-10 levels. Here, although cytokines (combined results) pointed out to an inflammatory effect, we unobserved an increasing in cell death. This phenomenon could be associated with the tested concentrations, since it was able to induce an increasing in cytokines levels, but still insufficient to cause death in tested cells (non-cytotoxic concentrations). This modulatory profile may be helpful in inflammatory conditions or in response against pathogens and tumors. However, in physiological condition, it could set an imbalance on immune system homeostasis inducing allergic events [22, 23]. Furthermore, this increase in secretion of proinflammatory cytokines could be promoting the DNA damage observed in our study. In fact, according to Bastos et al. 2017, inflammatory responses against infection or tissue injury could promote DNA damage in the form of chromosomal fragmentation, mutations points, and the formation of DNA adducts [24].

We suggest that the immunoregulatory effect can be promoted by NK cell activation according as demonstrate for Chen et al. 2006 in their study with γ- dodecalactone, which presents a similar chemical structure to γ-hexalactone. Nonetheless, complementary in vitro and in vivo investigations on immunological biomarkers could help us to validate the results and confirm the impact these data on immune system [25]. Thus, although the use of this flavoring is permitted, it is necessary to develop more studies to establish its safe concentrations mainly such as food flavoring [21, 22, 26].

## Conclusion

We have shown for the first time that γ-hexalactone has immunomodulatory potential and causes DNA damage in human lymphocyte at non-cytotoxic concentrations. Further studies should be performed to confirm the proposed mechanisms and to define the of safe concentrations range for use in food.

## Data Availability

All data generated or analyzed during this study are included in this published article.

## Abbreviations

ANOVA: One-way analysis of variance; NC: negative control
FAO: Food and Agriculture Organization
FBS: Fetal bovine serum
IC_50_: 50% cell-growth inhibition
JECFA: Joint FAO/WHO Expert Committee on Food Additives
PBMC: Peripheral blood mononuclear cells
PBS: Phosphate buffered saline
PHA-M: Phytohemagglutinin-M
WHO: World Health Organization
